# High-temperature superconductor of sodalite-like clathrate hafnium hexahydride

**DOI:** 10.1038/s41598-021-95112-5

**Published:** 2021-08-12

**Authors:** Prutthipong Tsuppayakorn-aek, Nakorn Phaisangittisakul, Rajeev Ahuja, Thiti Bovornratanaraks

**Affiliations:** 1 Extreme Conditions Physics Research Laboratory (ECPRL) and Physics of Energy Materials Research Unit, Department of Physics, Faculty of Science, Chulalongkorn University, Bangkok, 10330 Thailand; 2 Thailand Centre of Excellence in Physics, Ministry of Higher Education, Science, Research and Innovation, 328 Si Ayutthaya Road, Bangkok, 10400 Thailand; 3 Condensed Matter Theory Group, Department of Physics and Materials Science, Uppsala University, Box 530, Uppsala, SE 751 21 Sweden; 4 Department of Physics, Indian Institute of Technology (IIT) Ropar, Rupnagar, Punjab 140001 India

**Keywords:** Phase transitions and critical phenomena, Physics, Condensed-matter physics, Superconducting properties and materials

## Abstract

Hafnium hydrogen compounds have recently become the vibrant materials for structural prediction at high pressure, from their high potential candidate for high-temperature superconductors. In this work, we predict $\hbox {HfH}_{6}$ by exploiting the evolutionary searching. A high-pressure phase adopts a sodalite-like clathrate structure, showing the body-centered cubic structure with a space group of $Im\bar{3}m$. The first-principles calculations have been used, including the zero-point energy, to investigate the probable structures up to 600 GPa, and find that the $Im\bar{3}m$ structure is thermodynamically and dynamically stable. This remarkable result of the $Im\bar{3}m$ structure shows the van Hove singularity at the Fermi level by determining the density of states. We calculate a superconducting transition temperature ($T_{c}$) using Allen-Dynes equation and demonstrated that it exhibits superconductivity under high pressure with relatively high-$T_{c}$ of 132 K.

## Introduction

Hydrogen–rich materials at high pressure can achieve high-temperature superconductivity because of their outstanding hydrogen properties^1^, Aschroft further proposed that metallic alloys of heavier elements in hydrides besides hydrogen can considerably increase the electron-phonon coupling (EPC). Following this, the heavier elements reduce the pressure required for metallization through chemical pre-compression. Based on the Bardeen-Cooper-Schrieffer (BCS) theory, a high-temperature superconductor can be obtained from a phonon mediated superconductivity because it can open the way to extensive both experimental and theoretical researches^2–7^. In order to investigate the superconductive properties of metal hydrides, their crystal structures is a crucial information for the study^7–18^. For instance, $\hbox {LaH}_{10}$ was reported that it is a face-centered cubic structure with a space group of $Fm\bar{3}m$, and later on showing that it has a $T_{c}$ above 250 K^19,20^, besides, this material successfully demonstrated the importance of metallic hydrogen, appearing that it has a high potential for superconductivity. Using *ab* *initio* calculations, $\hbox {LaH}_{10}$ proposed to be an anharmonic phase because of the quantum effects, leading to reduce pressure for stabilize the structure^20^.

Room temperature superconductor is another holy grail in high-pressure physics, there are several materials which posses high-temperature superconductivity. Among those high promising materials, hydrogen-rich materials emerging as a vibrant candidate^7,14,21–27^. This important feature of metallic hydrogen has proved to be a dominant component for route to high-temperature superconductor. At present, the advancement of the room temperature superconductors can be obtained by materials design. For example, in metal hydrides, $\hbox {MgH}_{6}$ was theoretically studied by calcium substitution^7^. It found that $\hbox {Mg}_{0.5}\hbox {Ca}_{0.5}\hbox {H}_{6}$ is thermodynamically stable at high pressure, showing that the $T_{c}$ of $\hbox {Mg}_{0.5}\hbox {Ca}_{0.5}\hbox {H}_{6}$ is estimated to be 288 K at a pressure of 200 GPa. Another example of metal hydrides, hydrogen sulfide investigated the $T_{c}$ at high pressure. This work used $\hbox {CH}_{4}$ molecular to place into the bcc-$\hbox {H}_{3}\hbox {S}$ structure, leading to a magnificent discovery of high-$T_{c}$ from 100 K to 190 K at high pressure^28^. In the last example, Li-Mg-H compound predicted to be $\hbox {Li}_{2}\hbox {MgH}_{16}$^24^, and its $T_{c}$ predicted to be 423 K at a pressure of 250 GPa by increasing electron density of states at the Fermi level. These methodologies can point out that materials design can open a door for the possibility of achieving high-$T_{c}$.

Recently, the superconductivity of metal superhydrides was studied in a binary compound hafnium-hydrogen^29^, it can see hydrogen pentagraphenelike structure, which stabilized by hafnium. Following this case, the hydrogen pentagraphenelike structure is thermodynamically stable by hafnium. The remarkable result showed that a value of $T_{c}$ is around 213–234 K at a pressure of 250 GPa. The solution of this novel structure opened the door to the exploration of a new class of structure. Interestingly, it is worth to note that this work reported an energy difference between $\hbox {HfH}_{6}$ and $\hbox {HfH}_{10}$ which is closed by approximately 1-2 meV at a pressure of 300 GPa. The high pressure phase of $\hbox {HfH}_{6}$ is predicted to be a $Cmc2 _{1}$ structure^30,31^ and found that it is stable structure among a convex hull diagram. Moreover, the $Cmc2_{1}$ structure is reported to be dynamically stable at a pressure of 300 GPa^31^ because it does not indicate any imaginary frequency. Also, the value of $T_{c}$ of $\hbox {HfH}_{6}$ is estimated to be 45.2 K to 55 K. However, there are neither experimentally nor theoretically studies under high-pressure above 300 GPa.

It is interesting to note that transition metal hexahydride is thermodynamically and dynamically stable, as being in accordance with the high-$T_{c}$ such as $\hbox {ScH}_{6}$, $\hbox {YH}_{6}$, and $\hbox {ZrH}_{6}$, respectively. Among the predicted the value of $T_{c}$, based on the Allen–Dynes equation^32^. In 2017, $\hbox {ScH}_{6}$ was predicted the high-$T_{c}$ above 100 K from 300 to 400 GPa^33^. In the same year, $\hbox {ScH}_{6}$ was investigated by using the first-principles calculations, carried out the McMillan formula with Allen-Dynes corrections^32,34^. As result of this, $\hbox {ScH}_{6}$ displayed superconductivity with $T_{c}$ of 130 K at 285 GPa. Then, in 2018, $\hbox {ZrH}_{6}$ was explored the $T_{c}$, resulting in the estimation $T_{c}$ to be 114 K at 295 GPa^35^. Recently, in 2019, $\hbox {YH}_{6}$ was determined by using fully anisotropic Migdal-Eliashberg theory. The results on superconducting properties of $\hbox {YH}_{6}$ manifested the $T_{c}$ reads 290 K at 300 GPa^23^. Motivated by the prediction of $T_{c}$ of transition metal hexahydride, it is worthy to further explore $\hbox {HfH}_{6}$ at very high compressed conditions.

In this work, we provide a potential high pressure candidate structure of $\hbox {HfH}_{6}$, leading to scientific leap frog of high pressure superconductivity. We explore the high-pressure phase of $\hbox {HfH}_{6}$ under pressure from 300 GPa to 600 GPa by first-principles evolutionary techniques. Moreover, we aim to predict the value of $T_{c}$ by performing a candidate structure of $\hbox {HfH}_{6}$. Regarding its potential for superconductivity, the electronic properties shown to propound a possibility of the value of $T_{c}$ such as a band structure, a density of states, and a nature of chemical bonding. Particularly, the electronic properties play an important role in support the value of $T_{c}$.

## Methods

The searching for the structures of the clathrate hafnium hexahydride $\hbox {HfH}_{6}$ was performed by the Universal Structure Predictor: Evolutionary Xtallography (USPEX)^36^. In all subsequent generations, the random symmetric algorithm employed $40\%$ heredity, $20\%$ random symmetric, $\hbox {20}\%$ soft mutation, and $20\%$ transmutation operators in the pressure range from 200 to 600 GPa with structures containing up to four formula units. A plane-wave basis set up to cutoff energy of 700 eV and an initial Brillouin-zone (BZ) sampling grid of spacing $2\pi \times 0.02\,\mathring{A}\,^{-1}$ were used for this calculation as well as a plane-wave basis set up to cutoff is guaranteed to be converged within an accuracy of 3 meV per atom. All structures were fully relaxed using the generalized gradient approximation of the Perdew–Burke–Ernzerhof (GGA-PBE) functional^37^ for the exchange-correlation functional. We used the projector augmented wave (PAW) method^38^ and the conjugate gradient scheme, as implemented in the Vienna ab initio simulation package (VASP)^39^. For electron-phonon and the spectral function calculations, a plane-wave energy cutoff of 80 Ry was used. The dense k-points mesh contained all k and k+q grid points were used. The subsequent electron-phonon and spectral function calculations depended on the k-point part due to it covered the grid of q-point. The calculations were computed in the first BZ on $24 \times 24 \times 24$ k-points mesh and $2\times 2 \times 2$ q-meshes, showing that it is sufficient to produce accurate electron-phonon coupling. Computational details of the electron-phonon and spectral function calculations were successfully reported in the theoretical studies^16,17^ The Allen-Dynes equation^32^ was exploited with the effective Coulomb pseudopotential parameter, $\mu ^{*}= 0.10$.

as follows:

1 $$\begin{aligned} T_{c} = \frac{\omega _{log}}{1.2} \exp \Big [ -\frac{1.04(1+\lambda )}{\lambda -\mu ^*(1+0.62\lambda )} \Big ], \end{aligned}$$

where $\omega _{log}$ is the logarithmic average of the spectral function. $\lambda$ is the total electron-phonon coupling strength. The projected crystal orbital Hamilton population^40^ (pCOHP) used to explain the chemical bonding of the sodalite-like clathrate hafnium hexahydride structure, as implemented in LOBSTER code^41^.

## Results and discussion

Regarding ground-state structure in $\hbox {HfH}_{6}$, we aimed to identify the unknown structure of $\hbox {HfH}_{6}$ above 300 GPa due to theoretical predictions is a crucial key to the exploration of a candidate structures at high pressure. First of all, we predicted the high-pressure phase using USPEX code, it shows that our main structural prediction revealed low-enthalpy structures, showing an orthorhombic structure with a space group of $Cmc2 _{1}$ and a body centered cubic with a space group of $Im\bar{3}m$.

**Figure 1:**
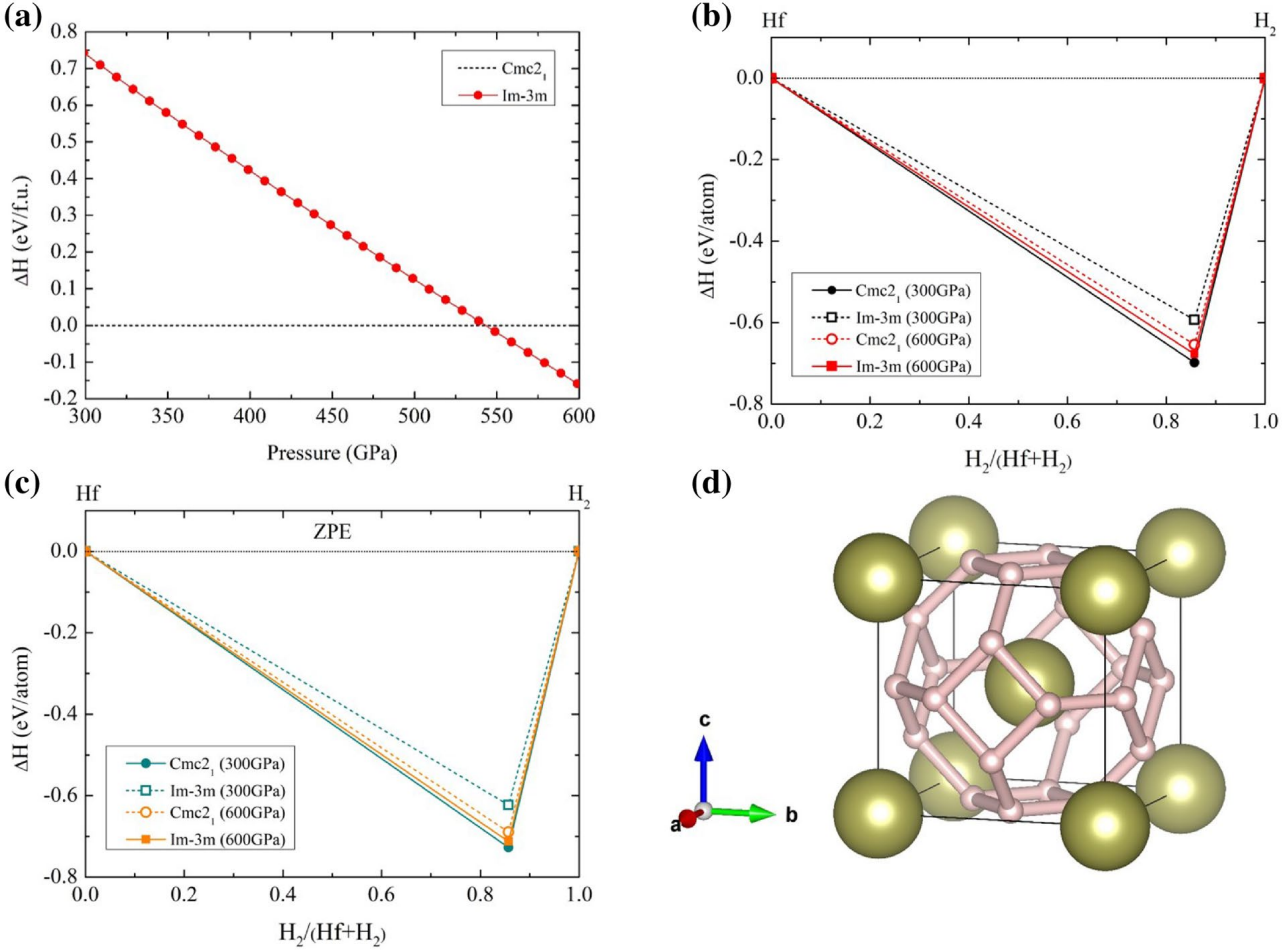
(**a**) The relative enthalpy of $\hbox {HfH}_{6}$ ranging from 300 to 600 GPa (**b**) Formation enthalpies of predicted $\hbox {HfH}_{6}$, excluding ZPE with respect to decomposition into Hf and H under pressure. (**c**) Formation enthalpies of predicted $\hbox {HfH}_{6}$, including ZPE with respect to decomposition into Hf and H under pressure. (**d**) The body-centred cubic structure of $\hbox {HfH}_{6}$, where the gold spheres represent the Hf atoms ans the pink sphere represents the H atoms, respectively.(drawn by VESTA (ver. 3.4.7)^51^ (URL https://jp-minerals.org/vesta/en/download.html)).

**Figure 2:**
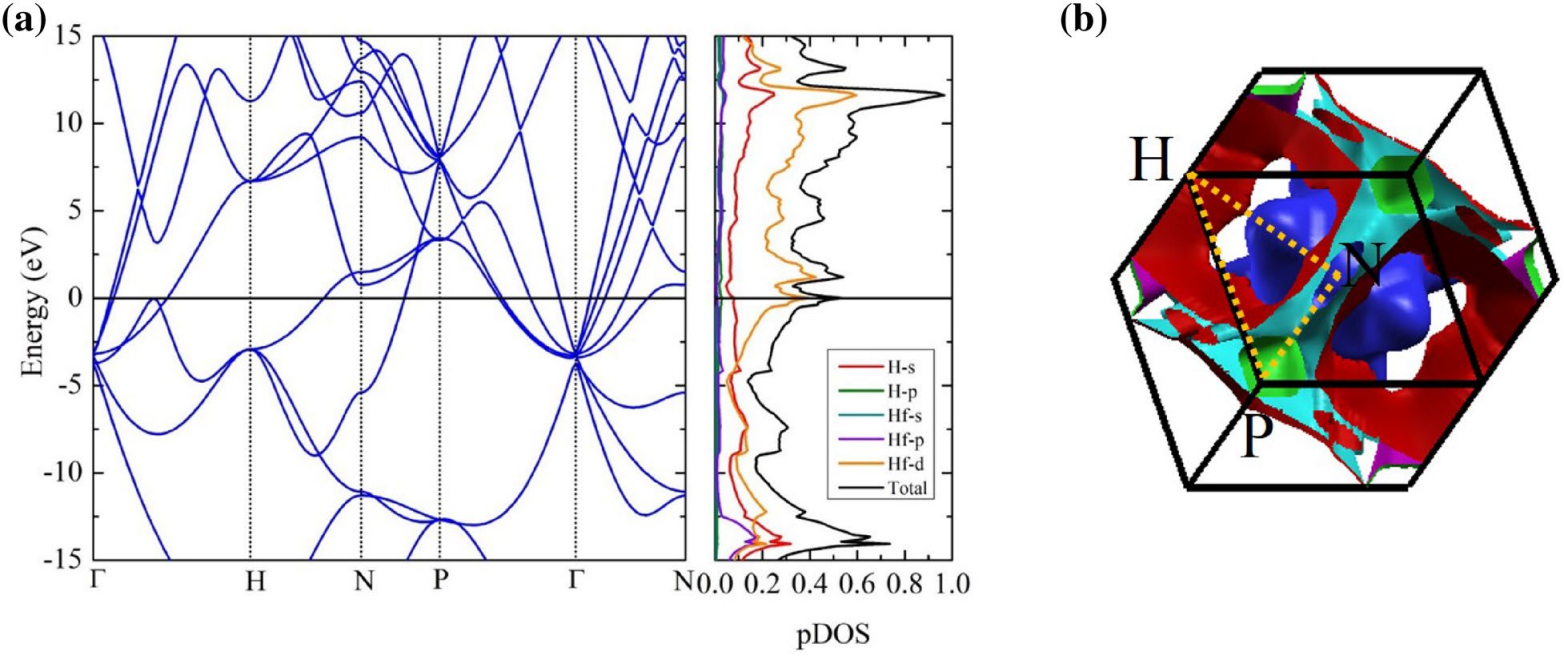
(**a**) The calculated electronic band structure and projected density of states of the sodalite-like clathrate hafnium hexahydride structure at 600 GPa. (**b**) Fermi surfaces of the sodalite-like clathrate hafnium hexahydride structure at 600 GPa. (drawn by XCrySDen program (ver. 1.5.60)^52^ (URL http://www.xcrysden.org/Download.html#_toc_1)).

For the first step in the structural predictions, a structural sequence showed that the $Cmc2 _{1}$ structure transformed into the $Im\bar{3}m$ structure at a pressure of 543 GPa. Under higher pressure, it found that the $Im\bar{3}m$ structure declined steadily up to 600 GPa, as showed in Fig. 1a. Moreover, we analyzed the further stabled structure of $\hbox {HfH}_{6}$ with respect to the elemental hafnium (the $Im\bar{3}m$ structure) and hydrogen (the *Cmca*-12 structure). Considering the relative enthlapy, one can see that the $Cmc2 _{1}$ structure is thermodynamically stable favored over the $Im\bar{3}m$ structure at a pressure of 300 GPa. On further compression to 600 GPa, the $Im\bar{3}m$ structure is apparently stable (Fig. 1b). Following this, we furture our investigation to the structural stability by the incorporation of the zero-point energy (ZPE) of the nuclei estimation, indicating that the $Im\bar{3}m$ structure is thermodynamically stable throughout the whole studied pressure range, as showed in Fig. 1c. It should be mentioned that our calculations performed the DFT at 0 K, we therefore investigated by considering at elevated temperatures. As a result, the $Im\bar{3}m$ structure is thermodynamically more stable than the $Cmc2 _{1}$ structure with increasing temperature up to at least 300 K, depicting in the convex hull envelopes at a pressure of 600 GPa of Fig. S1 in the Supplemental Material. This further implies the $Im\bar{3}m$ structure probably occurs at room temperature. Furthermore, we investigated further study of the stable structure of $\hbox {HfH}_{6}$ at a pressure of 300 GPa. As a result, we pointed out that the $Cmc2 _{1}$ structure is a potential candidate. Our calculations are in good agreement with those recently reported in the theoretical works^30,31^.

Here, we introduce sodalite-like clathrate at extremely high-pressure, showing the stabled bcc with the $Im\bar{3}m$ space group. To further describe this structure, the H atoms which is in the form of a sodalite-like cage, composing of eight H-hexagons and six H-squares, and Hf atoms crystallize into a lattice site of body-centered cube. The structural morphology showed in Fig. 1d, which resembles the structures of $\hbox {MgH}_{6}$^9^, $\hbox {CaH}_{6}$^8^ and $\hbox {YH}_{6}$^23^.

For the electronic property in the $Im\bar{3}m$ structure, it is clearly demonstrated in Fig. 2a. The band structure manifested a metallic state because a conduction band and a valence band crossed at the Fermi level. Besides, we found that the density of states (DOS) exhibited van Hove singularities (vHs) at the Fermi level, indicating a large electron-phonon coupling (EPC). Interestingly, it is worth noting that the vHs is dominated by a d-electron of Hf. As depicted in the DOS, the characteristics of the vHs play an important role in superconductivity. For example, $\hbox {H}_{3}\hbox {S}$^42,43^, $\hbox {YH}_{6}$^23^, and $\hbox {LaH}_{10}$^44^ systems, leading to the possibility of achieving high values of $T_{c}$. To further explore the electronic structure, the Fermi surface is described, as shown in Fig. 2b. It can see that the Fermi surfaces around the P-point exhibited the Fermi nesting because several Fermi surfaces are parallel to each other. It can thus enhance the EPC and the value of $T_{c}$.

**Figure 3:**
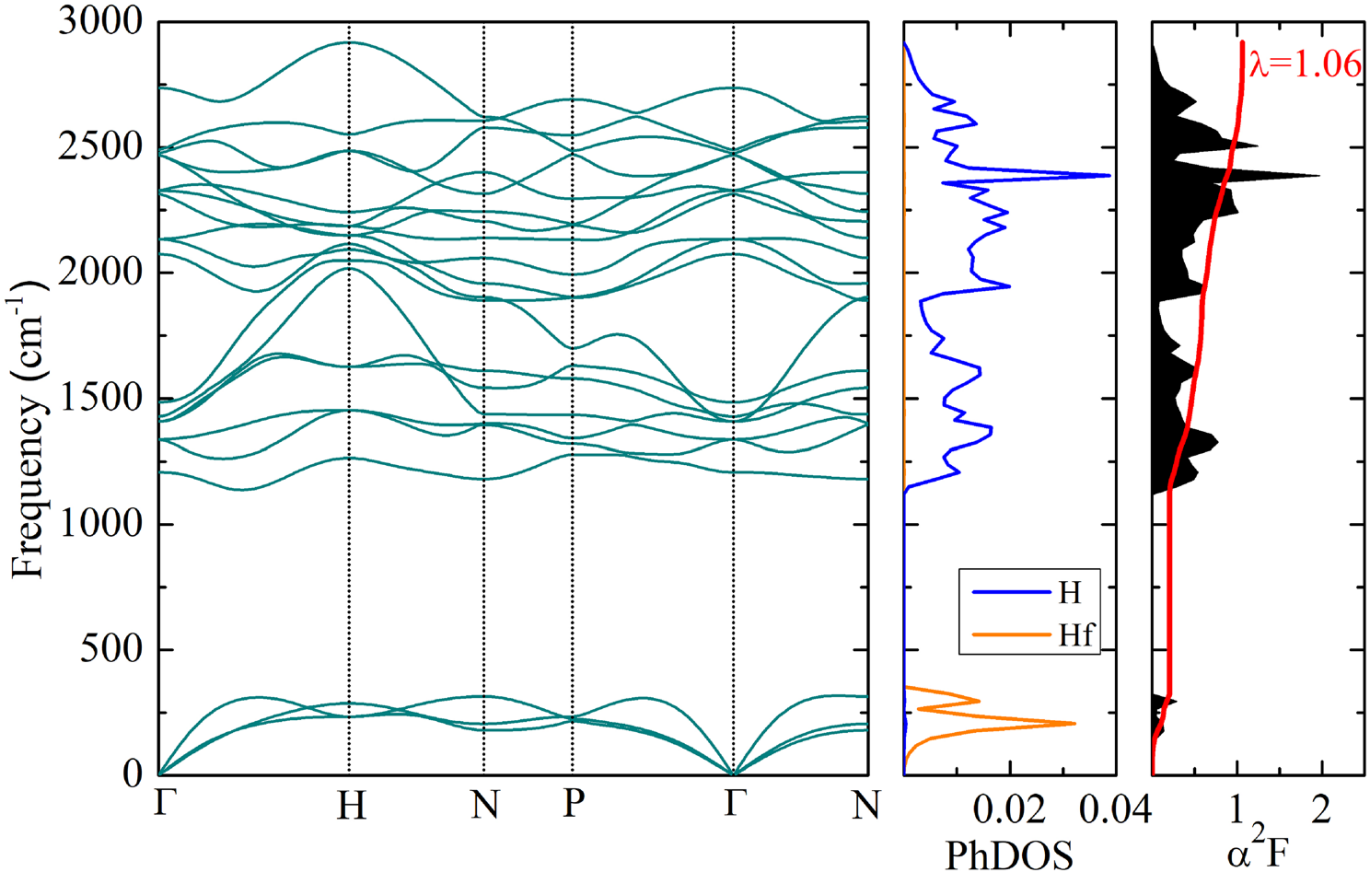
(Left) The calculated phonon dispersion at 600 GPa. (middle) The calculated projected phonon density of states at 600 GPa. (rigth) The Eliashberg spectral function and the integrating of lambda at 600 GPa.

**Figure 4:**
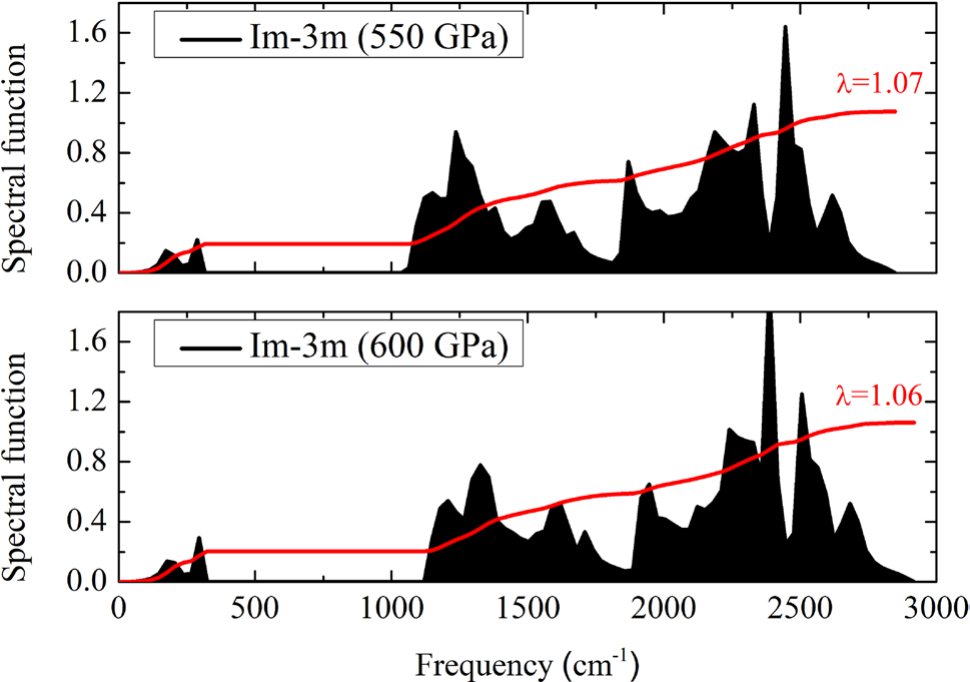
The Eliashberg spectral function and the integrating of lambda are calculated at 550 GPa and 600 GPa in the sodalite-like clathrate hafnium hexahydride structure.

According to Fig. 1, we computed phonon dispersions and phonon density of states (PhDOS) of the $Im\bar{3}m$ structure at a pressure of 600 GPa. As a result, we found that the $Im\bar{3}m$ structure is dynamically stable because it does not exhibit the imaginary frequency. Also, the phonon dispersions displayed acoustic modes and optical modes, as can be seen from Fig. 3, where the acoustic modes are the vibrations of the Hf atom and the optical modes are the vibrations of the H atoms. Moreover, the optical branches showed that there was an abundantly spread, showing the stretch and bent modes. These vibrations associated with the electron-phonon interaction and it yielded the high-$T_{c}$. Also, these characteristics corresponded with the PhDOS. It is interesting to note that the H atoms exhibited large vibrations by approximately from 1138 to 2918 THz. A remarkable solution is shown to propound a possibility of the high-$T_{c}$,as will be discussed later.

The spectral function $\alpha ^{2}F$ of the $Im\bar{3}m$ structure is calculated at a pressure of 600 GPa, as shown in Fig. 3. The Allen–Dynes equation^32^ carried out for the estimation $T_{c}$. It showed that the Eliashberg spectral function contributed slightly by approximately 0 $\hbox {cm}^{-1}$ to 324 $\hbox {cm}^{-1}$ and it contributed mainly by approximately 1120 $\hbox {cm}^{-1}$ to 2918 $\hbox {cm}^{-1}$. The solution of the integrating of lambda displayed that it climbed dramatically from 147 $\hbox {cm}^{-1}$ to 249 $\hbox {cm}^{-1}$. After that, it remained stable between 247 $\hbox {cm}^{-1}$ and 1149 $\hbox {cm}^{-1}$. Then, it increased moderately up to 2918 $\hbox {cm}^{-1}$, showing the integrating of lambda is 1.06. Here, we found that $\omega _{log}$ is 1741 K and the $T _{c}$ is 132 K, using $\mu ^{*}= 0.10$. Additionally, the $T_{c}$ is estimated by directly solving the McMillan formula with Allen-Dynes corrections $\mu ^{*} = 0.13$^34^. The calculated result shows that the estimated $T _{c}$ is 114 K. As a possible cause of this, one might think that the H atoms contributed a large frequency. Here again, we have already mentioned the DOS, it can see that an s-electron of H showed a large contribution to the DOS in comparison to s and p-electron of Hf, showing that it supported the existence of the high-$T _{c}$. As a result of this, we suggested that the existence of an unforeseen the $Im\bar{3}m$ structure with remarkably high-$T _{c}$ can pave the way for further studies on the trend of the high-temperature superconductors.

**Figure 5:**
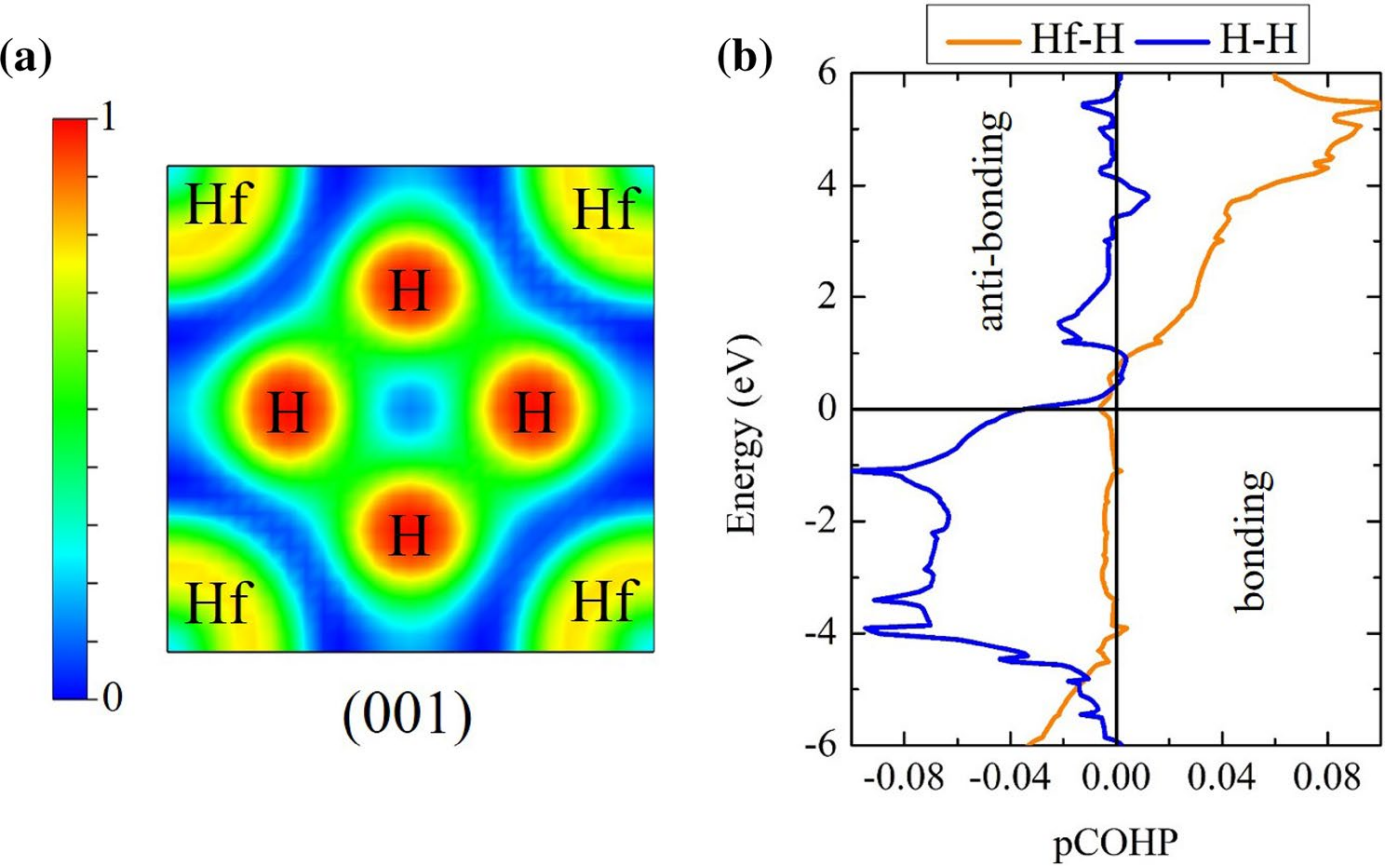
The 2D-electron localization function (ELF) in the sodalite-like clathrate hafnium hexahydride structure at 600 GPa (drawn by VESTA (ver. 3.4.7)^51^ (URL https://jp-minerals.org/vesta/en/download.html)). (**b**) Projected crystal orbital Hamilton populations (pCOHPs) in the sodalite-like clathrate hafnium hexahydride structure at 600 GPa.

To further analyse the spectral function of the $Im\bar{3}m$ structure, we calculated at a pressure of 550 GPa. Our calculations show that the character of the the spectral function is similar at a pressure of 600 GPa, as can be seen in Fig. 4. It exhibited the integrating of lambda is 1.07 and the $\omega _{log}$ is 1692 K, showing a high-$T _{c}$ of 130 K. At this point, as reported above, we found that the high-$T _{c}$ of the $Im\bar{3}m$ structure increased with increasing pressure. As a possible, one might think of the $\omega _{log}$. It showed that at a pressure of 600 GPa is the maximal of the $\omega _{log}$, which is higher than a a pressure of 550 GPa. We thus can point out that the $\omega _{log}$ plays an important role in the high-$T _{c}$ of $\hbox {HfH}_{6}$.

As mentioned earlier, it is also interesting to answer the question of why the $T _{c}$ of the $Im\bar{3}m$ structure is the high-$T _{c}$. At this point, we perform the electron localization function (ELF) and the projected crystal orbital Hamilton populations (pCOHP) solutions, the ELF method^45^ calculated to investigate bonding. The characteristics of ELF have successfully explained several materials^14,46–48^.

To begin with, the ELF of the $Im\bar{3}m$ structure is described a uniform electron gas of the same density in the (001) plane, as shown in Fig. 5a, it can be seen that a contribution of electrons between the H-H bonds are a weak bonding while the distribution of electrons in Hf atoms likely to be lone pairs in that region. Moving on to the pCOHP calculation, we described the character of the nature of a chemical bonding, which further supports the ELF calculation. This method can examine covalent bonding in several materials^11,49,50^. To further understand the superconductivity, the influence of bonding plays an important role in considering the value of $T_{c}$. The pCOHP calculation interprets the wave function into the covalent character. First of all, it can see that the H-H pairs promoted the anti-bonding. Following this, one can see that the Hf-H pairs were found to be the anti-bonding as well (Fig. 5). As a result of this, one might think that the nature of chemical bonding supported the value of $T _{c}$. This because the antibonding states in the covalent system led to the way of strong coupling of the EPC, which associated with the large vibration of H-rich.

## Conclusion

In this work, we identify the high-pressure phases of $\hbox {HfH}_{6}$ by performing an evolutionary searching. Overall, the incorporating of the zero-point energy shows that the $Im\bar{3}m$ structure is thermodynamically stable favored over the $Cmc2_{1}$ structure. The sodalite-like clathrate hafnium hexahydride is predicted to be a high-temperature superconductor with estimated $T_{c}$ of 132 K at a pressure of 600 GPa. The nature of the chemical bonding is associated with the electron localized function, implying that the characteristics of the chemical bonding entail the high-$T_{c}$. Finally, we point out that the existence of an unexpected the $Im\bar{3}m$ structure can pave the way for further studies on the development of the high-temperature superconductors.

## Supplementary information

Supplementary Information.

## Data Availability

The data that support the findings of this study are available from the corresponding author upon reasonable request.
