# Supplementary material for: High-temperature superconductor of sodalite-like clathrate hafnium hexahydride
Source: Sci Rep. 2021 Aug 12;11:16403. doi: 10.1038/s41598-021-95112-5 (PMC8361170; doi:10.1038/s41598-021-95112-5)
Supplement: Supplementary file 1 — Supplementary Information. [file 41598_2021_95112_MOESM1_ESM.docx]

Supplemental Materials

of

High-temperature superconductor of sodalite-likeclathrate hafnium hexahydride

Prutthipong Tsuppayakorn-aek^1,2^, Nakorn Phaisangittisakul^1,2^, Rajeev Ahuja^3,4^, and Thiti Bovornratanaraks^1,2,**^

^1^Extreme Conditions Physics Research Laboratory (ECPRL) and Physics of Energy Materials Research Unit, Department of Physics, Faculty of Science, Chulalongkorn University, Bangkok, 10330, Thailand

^2^Thailand Centre of Excellence in Physics, Ministry of Higher Education, Science, Research and Innovation, 328 SiAyutthaya Road, Bangkok 10400, Thailand

^3^Condensed Matter Theory Group, Department of Physics and Materials Science, Uppsala University, Box 530,SE-751 21, Uppsala, Sweden

^4^Department of Physics, Indian Institute of Technology (IIT) Ropar, Rupnagar 140001, Punjab, India

Corresponding author [**thiti.b@chula.ac.th](mailto:**thiti.b@chula.ac.th)

**Computational details**

The enthalpy calculation was calculated using the first-principles calculations, based on the density functional theory, as implemented in the VASP code [1] A plane-wave basis set up to cutoff energy of 700 eV and an initial Brillouin-zone (BZ) sampling grid of spacing 2π×0.02 Å^−1^ were used for this calculation. All structures were fully relaxed using the generalized gradient approximation of the Perdew–Burke–Ernzerhof (GGA-PBE) functional [2] for the exchange-correlation functional. The entropy is estimated within the quasi-harmonic approximation, where the frozen phonon method is carried out with the PHONOPY code [3], leading to the results of Gibbs free energy.

**Result and discussion**

Overall, the solution of the convex hulls calculations manifested that the formation enthalpies (non-ZPE and ZPE) are similar to the formation Gibbs free energy, as shown in Figure S1. We calculated the formation Gibbs free energy at temperature of 300 K. Yet, more significant unchanged in HfH_6_ at a pressure 600 GPa when the temperature is considered. Following this, we found that the Im-3m structure is thermodynamically stable favored over the Cmc2_1_ structure. This is evident in Figure S1 (c).


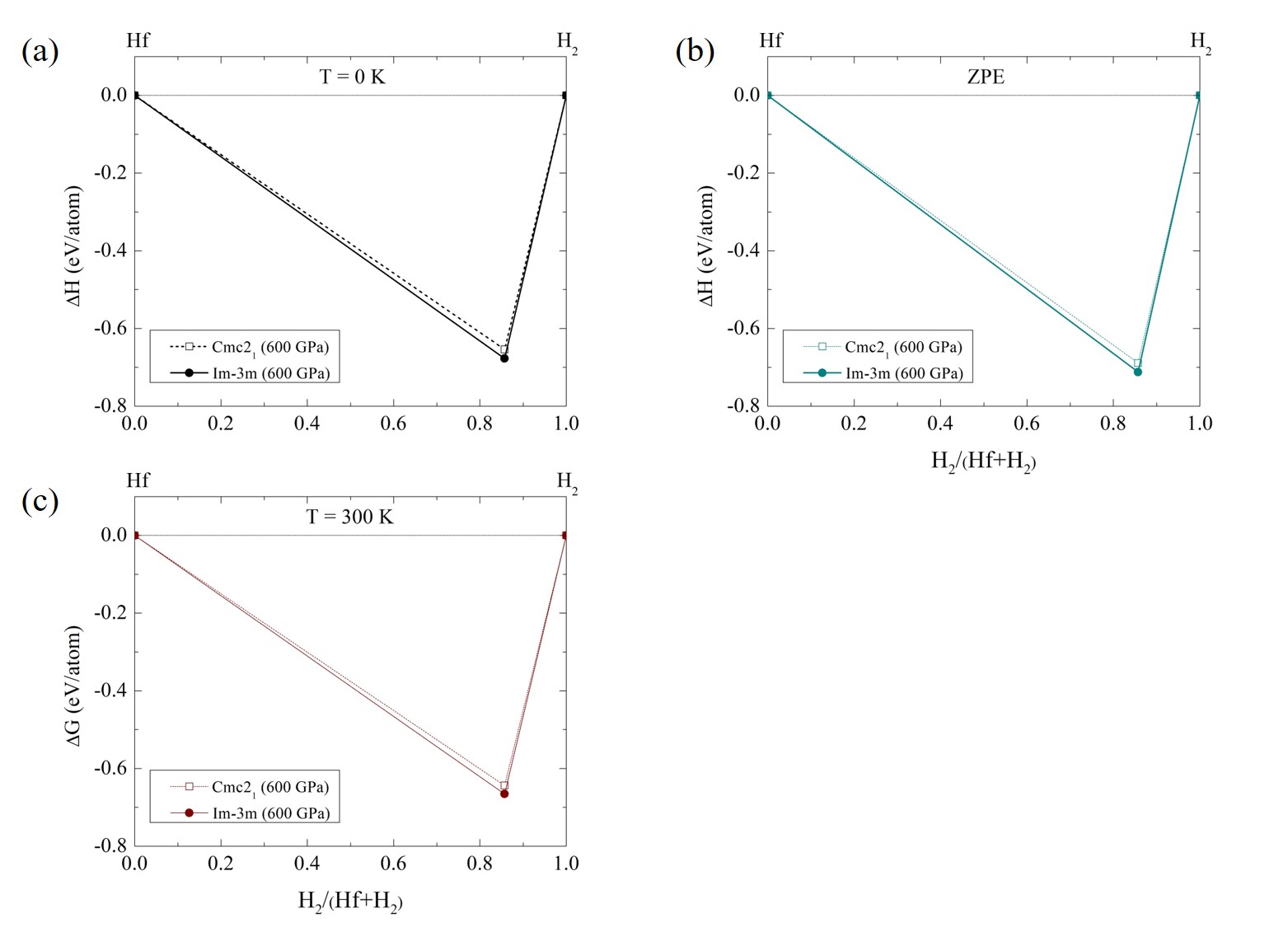


Figure S1: The convex hulls of HfH_6_ at a pressure of 600 GPa. (a) Formation enthalpies of predicted HfH_6_, excluding ZPE with respect to decomposition into Hf and H under pressure. (b) Formation enthalpies of predicted HfH_6_, including ZPE with respect to decomposition into Hf and H under pressure. (c) Formation Gibbs free energy of predicted HfH_6_ with respect to decomposition into Hf and H under pressure.

**References**

[1] Kresse, G. & Furthmüller, J. Efficient iterative schemes for ab initio total-energy calculations using a plane-wave basis set. Phys. Rev. B 54, 11169–11186 (1996).

[2] Perdew, J. P., Burke, K. & Ernzerhof, M. Generalized gradient approximation made simple. Phys. Rev. Lett.77, 3865–3868 (1996).

[3] Togo, A. & Tanaka, I. First principles phonon calculations in materials science. Scr. Mater. 108, 1–5 (2015).
